# Greater tuberosity fractures are not a continuation of Hill-Sachs lesions, but do they have a similar etiology?

**DOI:** 10.1016/j.jseint.2021.11.018

**Published:** 2022-01-12

**Authors:** Hassanin Alkaduhimi, Henk-Jan van der Woude, Lukas P.E. Verweij, Stein J. Janssen, Nienke W. Willigenburg, Neal Chen, Michel P.J. van den Bekerom

**Affiliations:** a Shoulder and Elbow Unit, Joint Research, OLVG, Amsterdam, the Netherlands; b Department of Orthopaedic Surgery, Amsterdam Movement Sciences, Amsterdam UMC, University of Amsterdam, Amsterdam, the Netherlands; c Academic Center for Evidence-based Sports Medicine (ACES), Amsterdam UMC, Amsterdam, the Netherlands; d Amsterdam Collaboration for Health and Safety in Sports (ACHSS), International Olympic Committee (IOC) Research Center, Amsterdam UMC, Amsterdam, the Netherlands; e Department of Hand and Upper Extremity Surgery, Massachusetts General Hospital, Boston, MA, USA; f Department of Human Movement Sciences, Faculty of Behavioural and Movement Sciences, Vrije Universiteit Amsterdam, Amsterdam Movement Sciences, Amsterdam, the Netherlands

**Keywords:** Shoulder, Instability, Glenohumeral, Greater tuberosity, Fracture, Hill, Sachs, Hill-sachs

## Abstract

**Background:**

It is unclear whether greater tuberosity fractures (GTF) in the setting of a shoulder dislocation are due to an avulsion of the rotator cuff or a result of an extensive Hill-Sachs lesion (HSL). To explore whether these lesions have similar etiology, the primary aim of this study is to compare the postinjury morphology of the proximal humerus after GTF and HSL.

**Methods:**

Computed tomography scans of 19 patients with HSL and 18 patients with GTF after first-time shoulder dislocations were analyzed. We assessed the location by measuring height in relation to the highest point of the humerus and angles for the origin (most medial point of lesion), center, and endpoint (most lateral point of lesion) between GTF and HSL and the bicipital groove. For both GTF and HSL, we assessed whether infraspinatus and supraspinatus insertions were involved and whether they were off-track or on-track.

**Results:**

Measured from the bicipital groove, HSLs and GTFs have different origins (153˚ vs. 110˚; *P* < .0001, respectively), centers (125˚ vs. 60˚; *P* < .0001, respectively), and endpoints (92˚ vs. 37˚; *P* < .0001, respectively). HSLs had a higher position (0.76 cm vs. 1.71 cm; *P* < .0001), involved the supraspinatus footprint less often (16% vs. 72%; *P* = .0008), and were less likely to be off-track (31% vs. 94%; *P* = .0002). Half of the GTF were on the lateral side of the glenoid track and thus extra-capsular, versus 0% of HSL.

**Conclusion:**

HSLs and GTFs have different anatomical characteristics and thus GTFs are likely to be distinct from extensive HSLs.

During an anterior shoulder dislocation the posterior part of the humeral head can collide with the glenoid resulting in a compression fracture of the posterolateral humeral head—commonly referred to as a Hill-Sachs lesion (HSL).^1^^,^^10^ An HSL is present in 40%-100% of glenohumeral dislocations^14^ and glenoid bone loss in 41%-86%.^6^ The glenoid track is the track representing the zone of contact between the glenoid and the humeral head and represents approximately 83% of the glenoid width.^6^ Di Giacomo et al^5^ have presented an off-track and on-track model to assess whether an HSL could cause additional instability. A lesion is called on-track if the HSL falls within the glenoid track and off-track when the medial margin of the HSL is outside the glenoid track resulting in no bone support of the glenoid and thus instability. ^5^

About 10%-30% of greater tuberosity fractures (GTFs) are due to a glenohumeral dislocation (Fig. 1).^8^ Traditionally, GTFs are described according to the Arbeitsgemeinschaft für Osteosynthesefragen (AO) classification or the Neer classification.^4^ A newer classification of Mutch et al^12^ has divided GTFs into 3 types. One of these types is a split fracture with a vertical fracture line, which is suggested to have a similar trauma mechanism as an HSL.^12^ Despite the suggested overlap in etiology, GTFs are associated with lower recurrence of shoulder instability compared to HSLs.^7^

**Figure 1:**
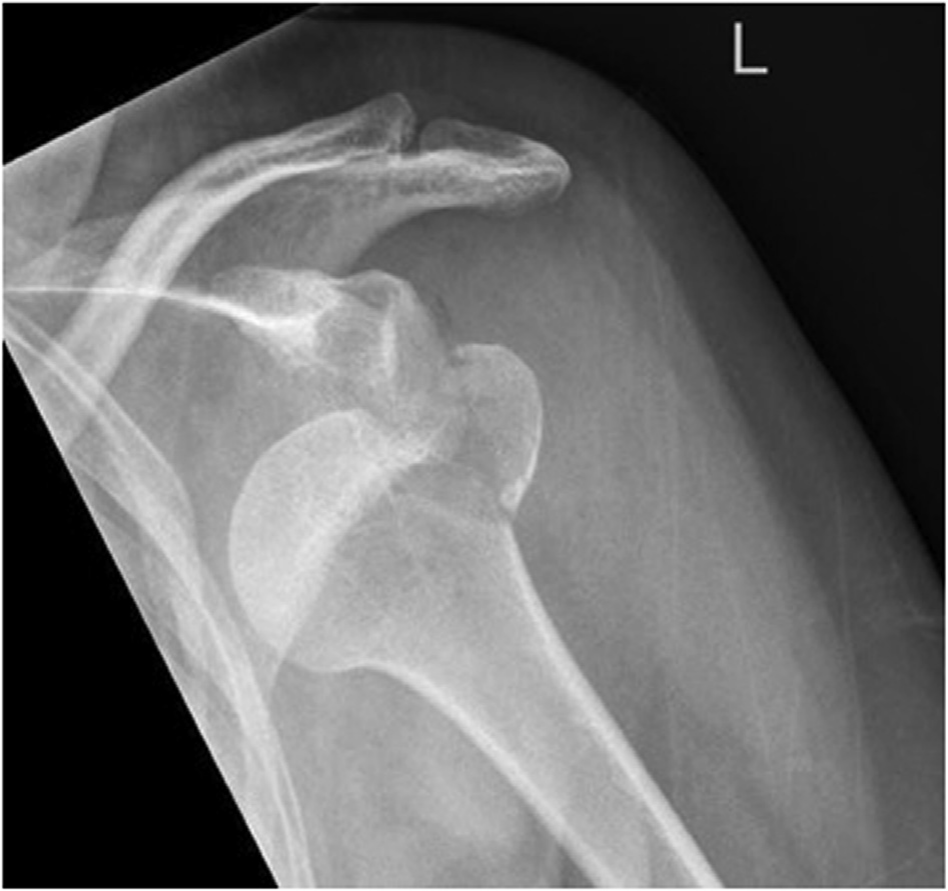
A shoulder dislocation with a concomitant greater tuberosity fracture.

To our knowledge, the differences in anatomical characteristics between GTFs and HSLs have not been reported. To explore whether these lesions could have similar etiology, the primary aim of this study is to compare the postinjury morphology of the proximal humerus after GTF and HSL. We hypothesize that HSLs and GTFs have similar anatomic features when described by location, involvement of the supraspinatus and infraspinatus tendon, and involvement of glenoid track.

## Methods

This study was performed according to the STrengthening the Reporting of OBservational studies in Epidemiology (STROBE) statement guidelines (Supplementary Appendix S1).

### Patient selection

The records of 2 large hospitals (OLVG in Amsterdam and Massachusetts General Hospital in Boston, MA, USA) between 2003 and 2013 were searched for computed tomography (CT) scans of patients after first-time shoulder dislocation with an HSL or a GTF. Furthermore, CT scans of patients aged <16 years, a CT slice thickness above 2.5 mm, other concomitant fractures, scans wherein the shoulder was still dislocated, and CT scans without an HSL or GTF were excluded. The sample size was based on expert opinion and the number of CT scans of GTF available in our hospitals. We predefined a minimum of 16 CTs per group, which is sufficient to detect differences in the magnitude of one standard deviation. Whether the lesion on the CT scan was classified as a GTF or HSL was dependent on the classification performed by the musculoskeletal radiologist (H.J.vW.). This was performed according to the morphological characteristics as proposed by Mutch et al.^12^

### Preparation of computed tomography scans

CT scan DICOM (Digital Imaging and Communications in Medicine) files were obtained through the radiology archiving system of the hospitals. Different CT scanners were used up to 120-140 Kv and 500-700 mA. For measurement of the location of the Hill-Sachs and the involvement of the rotator cuff footprint, we evaluated the DICOM files using RadiAnt DICOM viewer (Medixant, Poznan, Poland; RadiAnt DICOM Viewer [Software] URL: https://www.radiantviewer.com). We used OsiriX DICOM Viewer^15^ to render a 3-dimensional model of the CT scans and analyzed the glenoid track with Meshmixer (Autodesk Inc., San Rafael, CA, USA; www.meshmixer.com).

### Measurements

To determine the anatomical characteristics, we performed multiple measurements: the angle of the origin, center, and endpoint of the GTF and HSL in relation to the bicipital groove (bicipital angle), the height, involvement of the supraspinatus and infraspinatus tendon footprint, and whether the lesions are off-track or on-track. The first and fourth authors (H.A. and H.J.vW.) have measured the locations and the involvement of the supraspinatus and infraspinatus tendon simultaneously and discussed the measurements. The measurements agreed upon are presented. Measurements of the off-track and on-track concept were performed by the first and fifth author (L.P.E.V) and the agreed upon measurements are presented.

**Figure 2:**
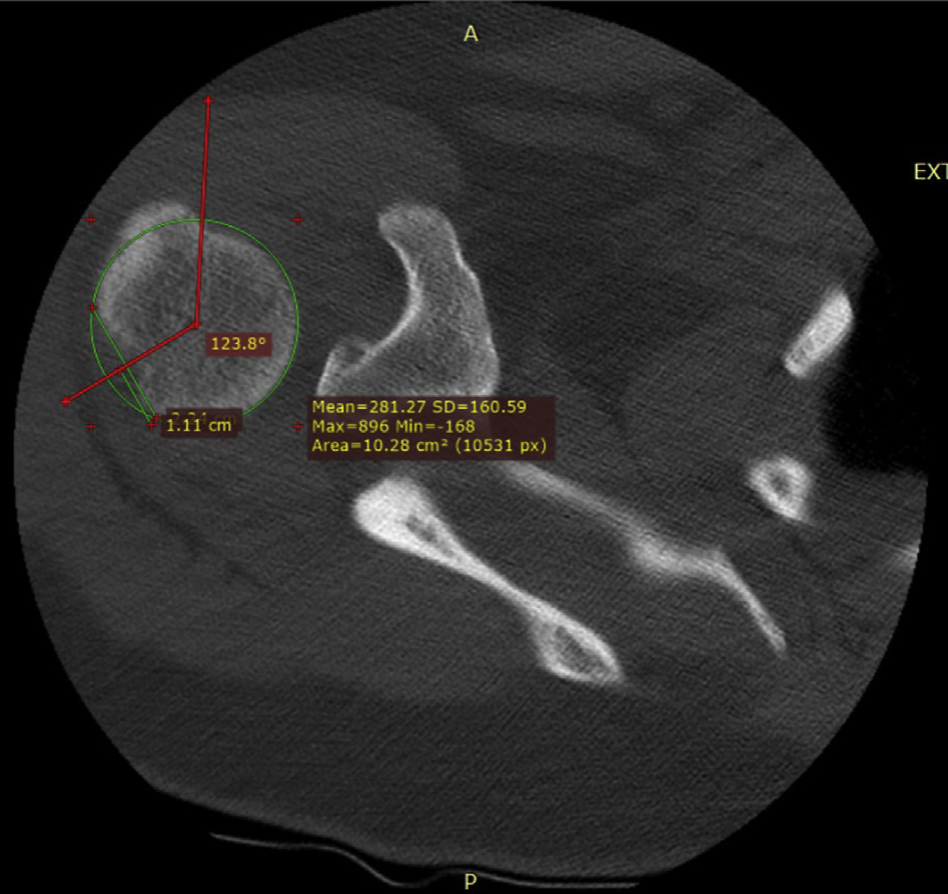
The bicipital angle of a Hill-Sachs lesion is determined. First, we draw a best-fit *circle* in line with the articular surface. Second, we determine the origin (most medial point of the HSL) and endpoint (most lateral point of the HSL). Third, we draw a line between the origin and endpoint. The midpoint of this line is the center. The bicipital angle for these points is the angle between the bicipital groove and these points.

**Figure 3:**
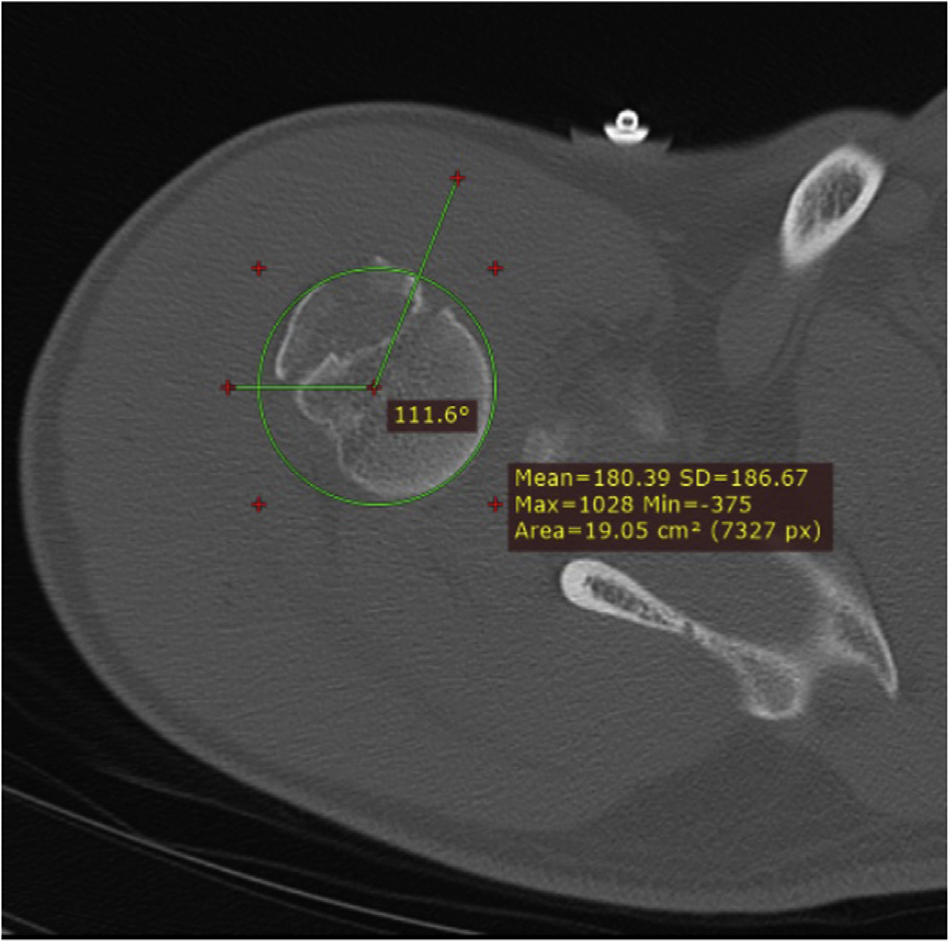
To measure the angle between the origin of the greater tuberosity fracture and the bicipital groove, we have drawn a *circle* in line with the articular surface of the humeral head. We have then measured the angle between the origin, center, and midpoint of the fracture and the bicipital groove according to the same steps as in Figure 2.

**Figure 4:**
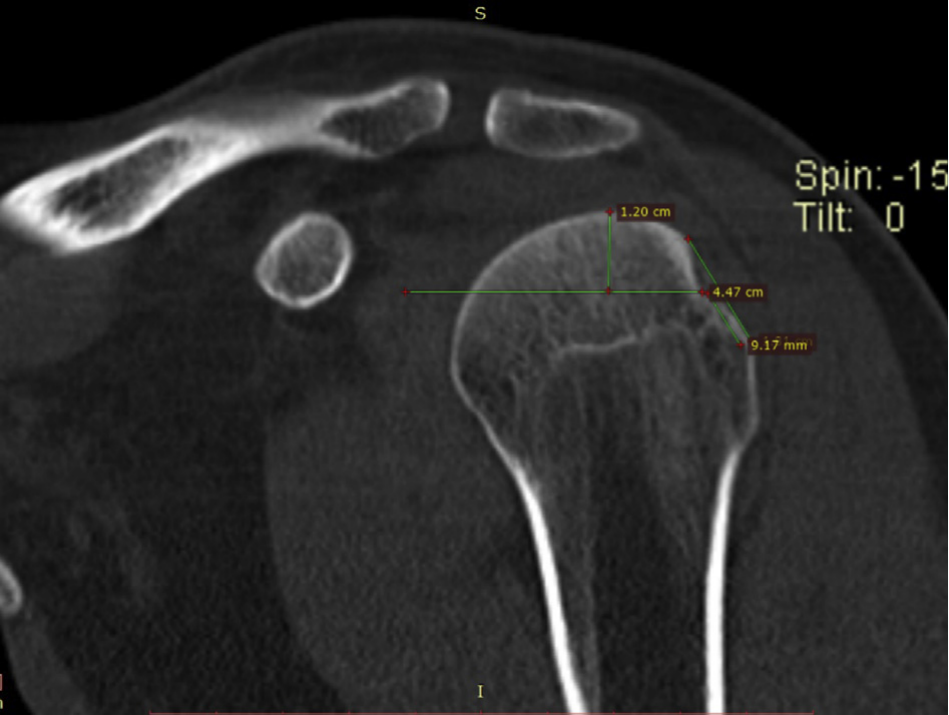
The height was measured by measuring the difference between the center of the lesions and the most cranial point of the humeral head.

The detailed explanation of the measurements is as follows. A best-fit circle was drawn in line with the articular surface of the humeral head on the axial plane. The most medial point of the HSL was defined as the origin and the most lateral point of the HSL was defined as the endpoint of the lesion. A line was drawn between the origin and endpoint, wherein the midpoint of this line was defined as the center of the HSL. We then measured the angle from the bicipital groove to the origin, center, and endpoint of the HSL. These are called the bicipital angles (Fig. 2). For the GTFs, we measured the origin, center, and endpoint in the same manner on the level of the coracoid process (Fig. 3). If the impact lines of the GTFs ended before reaching the level of the coracoid process, we measured the origin on the highest measurable level. The height of the HSL and GTF was determined in the sagittal plane by measuring the distance between the center of the lesion and the highest point of the humeral head (Fig. 4). Following these measurements, the bicipital angles of the origin, center, and endpoints for the HSL and GTFs were compared. Since theoretically the place of impact could be in the center of the HSL and at the origin of the GTF, we compared these 2 points as well.

Furthermore, we assessed whether the HSL and GTF involved the footprint of the supraspinatus and infraspinatus tendon. For these measurements, we used the locations described by Mochizuki et al.^11^

**Figure 5:**
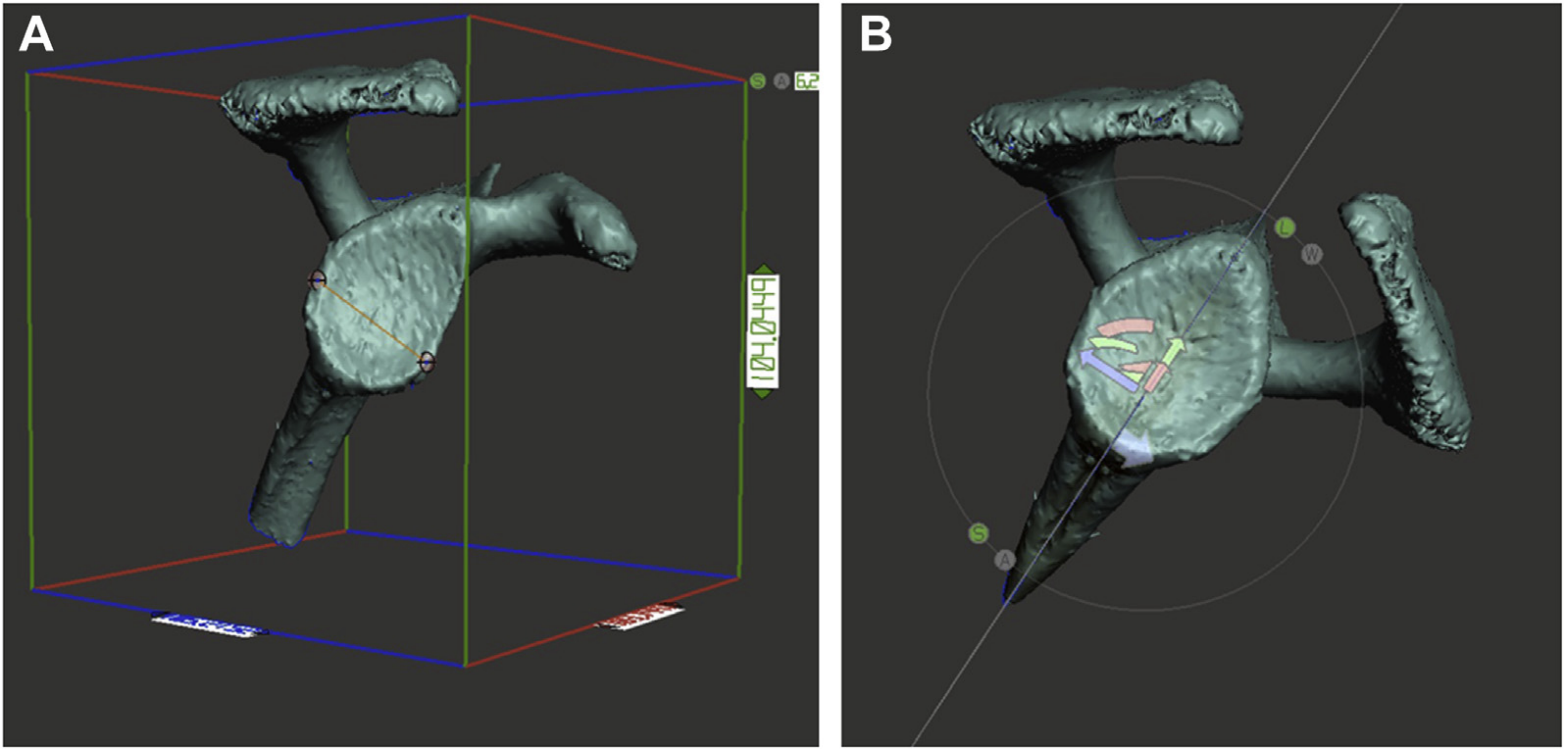
(**A**) The affected glenoid width is measured on an en face view of a 3-dimensional reconstruction of the CT. (**B**) The glenoid is mirrored symmetrically in order to measure the unaffected glenoid width.

**Figure 6:**
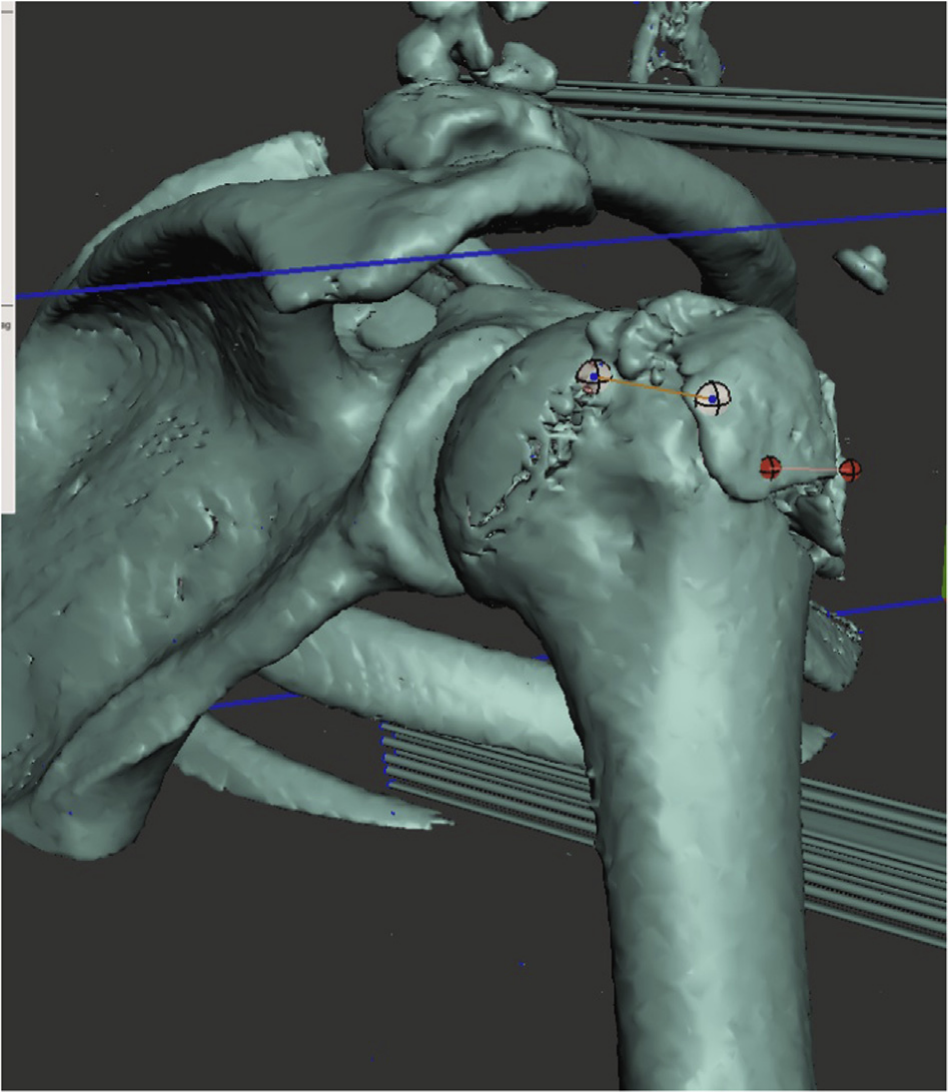
The width of the glenoid track is overlayed on to the humeral head from the medial margin of the rotator cuff footprint to the medial side () resembling the glenoid track. If the HSL was larger or located in a manner that exceeded the medial margin of the glenoid track, it was considered an off-track lesion. In this figure you can see a lateral greater tuberosity fracture that inserts the glenoid track, but is so lateral that it is extracapsular and most likely does not contribute to instability.

Finally, we assessed whether the HSL and GTF were on-track or off-track according to the following steps described by Di Giacomo et al.^5^ A 3-dimensional reconstruction of the shoulder was made. First, we measured the width of the affected glenoid on this reconstruction (Fig. 5, *A*). Second, since the inferior two-third of the glenoid approximates a true circle,^16^ we mirrored the glenoid to be able to simulate the unaffected glenoid. We then measured the width of the mirrored “unaffected” glenoid (Fig. 5, *B*). Third, we used the formula GT = 0.83*D*−*d* to calculate the glenoid track. Herein, *D* resembles the width of the glenoid and *d* is the width of the anterior glenoid bone loss (unaffected width minus affected width). Fourth, we overlayed the width of the calculated glenoid track on to the humeral head from the margin of the rotator cuff footprint to the medial side (Fig. 6). This resembles the glenoid track. If the HSL was larger or located in a manner that exceeded the medial margin of the glenoid track, it was considered an off-track lesion.

### Statistical analysis

To compare the angle in relation to the bicipital groove and the height of the HSL and GTF, we used Student’s *t*-test for normally distributed data and a Mann-Whitney *U*-test for non-normally distributed data. For the involvement of the infraspinatus and supraspinatus insertion and whether the lesions were on-track or off-track, we used a Fisher’s exact test. The level of significance was set at α= 0.05.

## Results

We identified 18 CT scans of patients with a GTF and selected the first 19 consecutive CT scans with an HSL. The patient records were analyzed retrospectively. In our cohort of CT scans, HSLs and GTFs did not co-exist in the same patient.

### Demographic characteristics

Table I summarizes the demographic characteristics. There were no statistical differences in age, gender, side of the affected arm, and injury mechanism. The average difference in age was 11 years, with substantial variation within the groups. Half of the GTFs occurred during a fall from standing, while 42% of the HSL occurred during sports. Other demographics were quite similar between groups.

**Table I:** Demographics of our study participants.

	HSL	GTF	*P* value
Age (yr) (mean ± SD)	39 ± 17	50 ± 16	.06
Male	12 (63%)	8 (44%)	.33
Right side	11 (58%)	8 (44%)	.52
Fall from standing	2 (11%)	9 (50%)	.08
Sports	8 (42%)	4 (22%)	
Seizure	1 (5%)	1 (6%)	
Mechanism			
Unknown	4 (21%)	1 (6%)	
Road traffic accident	1 (5%)	2 (11%)	
Fight/assault	1 (5%)	1 (6%)	
Atraumatic	2 (11%)	0 (0%)	

*HSL*, Hill-Sachs lesion; *GTF*, greater tuberosity fracture; *SD*, standard deviation.

### Location and height

The HSLs and GTFs have a different bicipital angle for the origin (153˚ vs. 110˚; *P* < .0001, respectively), center (125˚ vs. 60˚; *P* < .0001, respectively), and endpoint (92˚ vs. 37˚; *P* < .0001, respectively). In other words, GTFs are located more anteriorly and laterally and are larger in size (Table II). When comparing the bicipital angle of the center of the HSL with the bicipital angle of the origin of the GTF, we did not find significant differences (125˚ vs. 110˚; *P* = .05).

**Table II:** Results of the study.

Variable	Hill-Sachs	Greater tuberosity	*P* value
Angle of origin (°)	153	110	<.0001
Angle of center (°)	125	60	<.0001
Angle of end point (°)	92	37	<.0001
Height (cm)	0.76	1.71	<.0001
Involvement supraspinatus, n (%)	3 (16%)	13 (72%)	.0008
Involvement infraspinatus, n (%)	19 (100%)	18 (100%)	1
Off-track, n (%)	5 (31%)	16 (94%)	.0002
Involves lateral margin of glenoid track	0 (0%)	9 (50%)	.0004

The angle of origin, center, and endpoint and the height represent mean values.

The HSLs were located higher in the humeral head in comparison with GTFs (0.76 cm vs. 1.71 cm; *P* < .0001).

### Involvement of footprints and glenoid track

All HSLs and GTFs involved the footprint of the infraspinatus. GTFs were more likely to involve the supraspinatus footprint (72% vs 16%; *P* = .0008). The average *D* and d were not different between HSL and GTF (*D*: 26.7 vs. 27.4, *P* = .5167; d: 2.7 vs. 2.1, *P* = .15, respectively). GTFs were more likely to be off-track (94% vs. 31%; *P* = .0002) and were located more on the lateral side of the glenoid track. In 50% of the GTF cases the lateral margin of the glenoid track was involved, while the medial margin was intact. These lesions were thus mostly extracapsular. None of the HSLs involved the lateral margin of the glenoid track (*P* = .0004).

## Discussion

The results of this study show that GTFs and HSLs have different anatomical characteristics. The origin, center, endpoint, and height were different for these 2 lesions. GTFs were more likely to involve the supraspinatus footprint and more likely to be off-track.

A limitation of this study is that fracture mapping was not possible due to differences in lesion characteristics (eg, an HSL being an impression fracture and a GTF being a fracture with a displaced greater tuberosity). Therefore, we assessed the location of the HSLs and GTFs in the axial and sagittal plane separately, as described by Cho et al.^2^ Another limitation is that the relatively small sample size was sufficient to detect large (Cohen’s *d* ≈ 1) differences between groups.^3^ More subtle differences may also exist but did not reach statistical significance in this study. Furthermore, the glenoid track concept is not validated for GTF and thus these findings should be interpreted cautiously. Moreover, although all measurements were performed by 2 investigators, we know that interobserver reliability of CT scan measurements is limited. This could affect reproducibility of our findings. Finally, in our practices we did not perform CT scans for all patients. Since we only included patients with a CT scan retrospectively, this could lead to selection bias.

When comparing our findings with previous work on this topic, the bicipital angle in our study was a bit higher than reported by Cho et al^2^ (114.6˚ in engaging lesions and 118.8˚ in non-engaging lesions). Since we have followed the same measurement method in Cho et al, this could be due to measurement variation. This difference in absolute angles does not affect the main findings of this study comparing HSL and GTF. Hasan et al^9^ concluded that GTFs are more likely to occur in the zone of the interval between the supraspinatus and infraspinatus footprint. In our study, all GTFs involved the infraspinatus tendon footprint and 72% involved the supraspinatus footprint. This corresponds with the results of Hasan et al regarding that these lesions are mostly located in the interval between the supraspinatus and infraspinatus footprint.

GTFs are associated with a lower recurrent dislocation rate.^7^^,^^13^ Our results could explain this phenomenon as GTFs are more laterally oriented in comparison with HSL and thus are more likely to be extracapsular. A second explanation could be that due to the existence of the fracture less force is excreted to the glenoid rim and the capsulolabral structures. A third factor contributing to the lower recurrence rate could be the loss of end range of motion resulting in less engagement of the fracture with the glenoid.^7^ A fourth possible contributing factor could be that these lesions are associated with the middle aged population.^7^ Since age is a predicting factor for instability, these patients could be less prone to recurrent instability.^7^ This also results in protection from instability by preventing the patient from performing movements leading to instability in end range of abduction and external rotation.

Three different etiologies are postulated regarding HSLs and GTFs in the setting of glenohumeral dislocation:

1. GTFs and HSLs are both caused by impaction of the humeral head with the glenoid.
2. GTFs are a result of an avulsion of the rotator cuff.
3. GTFs are a combination of an avulsion and an impaction fracture; if the lateral side of the humeral head collides with the glenoid the impact results in a weak spot which aids the rotator cuff to avulse the greater tuberosity.

Our results show that HSLs and GTFs differ in location and therefore we believe that the moment of impact of GTFs and HSL is different. This makes theory 1 that they are both due to an impaction of the humeral head and glenoid unlikely. We observed that GTFs were laterally located. This corresponds with theory 2 that GTF may be a result of an avulsion fracture caused by the rotator cuff. Furthermore, it clarifies that fracture patterns are closely related to the rotator cuff attachments.^9^ The fact that there were no patients having both a GTF and an HSL supports the theory of a combination of an avulsion and an impaction fracture. In summary, it is likely that most GTFs are avulsion fractures of the rotator cuff.

## Conclusion

HSLs and GTFs have different anatomical characteristics and thus GTFs are likely to be distinct from extensive HSLs.

## Disclaimers

Funding: No funding was disclosed by the authors.

Conflicts of interest: The authors, their immediate families, and any research foundation with which they are affiliated have not received any financial payments or other benefits from any commercial entity related to the subject of this article.
