# Supplementary material for: Greater tuberosity fractures are not a continuation of Hill-Sachs lesions, but do they have a similar etiology?
Source: JSES Int. 2022 Jan 12;6(3):396–400. doi: 10.1016/j.jseint.2021.11.018 (PMC9091784; doi:10.1016/j.jseint.2021.11.018)
Supplement: Supplementary Appendix S1 [file mmc1.doc]

STROBE Statement—checklist of items that should be included in reports of observational studies

|  | Item No | Recommendation |
| --- | --- | --- |
| **Title and abstract** | 1 | (*a*) Indicate the study’s design with a commonly used term in the title or the abstract 🡪 *“Level of evidence: III, radiological study, abstract* |
|  |  | (*b*) Provide in the abstract an informative and balanced summary of what was done and what was found 🡪 *Structured abstract with background, methods, results, and conclusion* |
| Introduction | | |
| Background/rationale | 2 | Explain the scientific background and rationale for the investigation being reported 🡪 *“To our knowledge, the differences in anatomical characteristics between GTFs and HSLs have not been reported. To explore whether these lesions could have similar etiology, the primary aim of this study is to compare the post-injury morphology of the proximal humerus after GTF and HSL.”, page 3, line 76-79* |
| Objectives | 3 | State specific objectives, including any prespecified hypotheses *🡪 “To our knowledge, the differences in anatomical characteristics between GTFs and HSLs have not been reported. To explore whether these lesions could have similar etiology, the primary aim of this study is to compare the post-injury morphology of the proximal humerus after GTF and HSL. We hypothesize that HSLs and GTFs have similar anatomic features when described by location, involvement of the supraspinatus and infraspinatus tendon, and involvement of glenoid track.”, page 3, line 76-81* |
| Methods | | |
| Study design | 4 | Present key elements of study design early in the paper *🡪 Method section subcategorized in patient selection, preparation of CT scans, and measurements* |
| Setting | 5 | Describe the setting, locations, and relevant dates, including periods of recruitment, exposure, follow-up, and data collection *🡪 “The records of two large hospitals (OLVG in Amsterdam and Massachusetts General Hospital in Boston) between 2003 and 2013 were searched for CT scans of patients after first-time shoulder dislocation with a HSL or a GTF. Furthermore, CT-scans of patients aged < 16 years, a CT-slice thickness above 2.5mm, other concomitant fractures, scans wherein the shoulder was still dislocated, and CT scans without a HSL or GTF were excluded. The sample size was based on expert opinion and the number of CT scans of GTF available in our hospitals. We predefined a minimum of 16 CTs per group, which is sufficient to detect differences in the magnitude of one standard deviation. page 4, line 91-98* |
| Participants | 6 | (*a*) *Cohort study*—Give the eligibility criteria, and the sources and methods of selection of participants. Describe methods of follow-up  *Case-control study*—Give the eligibility criteria, and the sources and methods of case ascertainment and control selection. Give the rationale for the choice of cases and controls  *Cross-sectional study*—Give the eligibility criteria, and the sources and methods of selection of participants 🡪 *patient selection section in methods (see section 5)* |
|  |  | (*b*) *Cohort study*—For matched studies, give matching criteria and number of exposed and unexposed  *Case-control study*—For matched studies, give matching criteria and the number of controls per case *🡪 patient selection section in methods (see section 5)* |
| Variables | 7 | Clearly define all outcomes, exposures, predictors, potential confounders, and effect modifiers. Give diagnostic criteria, if applicable *🡪 section measurements and statistical analysis in methods* |
| Data sources/ measurement | 8* | For each variable of interest, give sources of data and details of methods of assessment (measurement). Describe comparability of assessment methods if there is more than one group *🡪 section measurements in methods* |
| Bias | 9 | Describe any efforts to address potential sources of bias *🡪 “A limitation of this study is that fracture mapping was not possible due to differences in lesion characteristics (e.g. a HSL being an impression fracture and a GTF being a fracture with a displaced greater tuberosity). Therefore, we assessed the location of the HSLs and GTFs in the axial and sagittal plane separately, as described by Cho et al.. 2 Another limitation is that the relatively small sample size was sufficient to detect large (Cohen’s d≈1) differences between groups. 3 More subtle differences may also exist but did not reach statistical significance in this study. Furthermore, the glenoid track concept is not validated for GTF and thus these findings should be interpreted cautiously. Moreover, although all measurements were performed by two investigators, we know that interobserver reliability of CT scan measurements is limited. This could affect reproducibility of our findings. Finally, in our practices we did not perform CT scans for all patients. Since we only included patients with a CT scan retrospectively, this could lead to selection bias..”, page 10, line 190-201* |
| Study size | 10 | Explain how the study size was arrived at *🡪 “The sample size was based on expert opinion and the number of CT scans of GTF available in our hospitals. We predefined a minimum of 16 CTs per group, which is sufficient to detect differences in the magnitude of one standard deviation.”, page 4, line 95-98* |
| Quantitative variables | 11 | Explain how quantitative variables were handled in the analyses. If applicable, describe which groupings were chosen and why *🡪 “To compare the angle in relation to the bicipital groove and the height of the HSL and GTF we used student’s t-test for normally distributed data and a Mann-Whitney U test for non-normally distributed data. For the involvement of the infraspinatus and supraspinatus insertion and whether the lesions were on-track or off-track we used a Fisher’s exact test”, page 6, line 123-126* |
| Statistical methods | 12 | (*a*) Describe all statistical methods, including those used to control for confounding *🡪 “To compare the angle in relation to the bicipital groove and the height of the HSL and GTF we used student’s t-test for normally distributed data and a Mann-Whitney U test for non-normally distributed data. For the involvement of the infraspinatus and supraspinatus insertion and whether the lesions were on-track or off-track we used a Fisher’s exact test.”, page 6-7, line 155-158* |
|  |  | (*b*) Describe any methods used to examine subgroups and interactions *🡪 “To compare the angle in relation to the bicipital groove and the height of the HSL and GTF we used student’s t-test for normally distributed data and a Mann-Whitney U test for non-normally distributed data. For the involvement of the infraspinatus and supraspinatus insertion and whether the lesions were on-track or off-track we used a Fisher’s exact test.”, page 6-7, line 155-158* |
|  |  | (*c*) Explain how missing data were addressed *🡪 due to the retrospective character of this radiological study we did not have missing data or loss to follow up* |
|  |  | (*d*) *Cohort study*—If applicable, explain how loss to follow-up was addressed  *Case-control study*—If applicable, explain how matching of cases and controls was addressed  *Cross-sectional study*—If applicable, describe analytical methods taking account of sampling strategy *🡪 due to the retrospective character of this radiological study we did not have missing data or loss to follow up* |
|  |  | (*e*) Describe any sensitivity analyses *🡪 A sensitivity analysis was not applicable in this study* |

Continued on next page

| Results | | |
| --- | --- | --- |
| Participants | 13* | (a) Report numbers of individuals at each stage of study—eg numbers potentially eligible, examined for eligibility, confirmed eligible, included in the study, completing follow-up, and analysed *🡪 “We identified 18 CT scans of patients with a GTF and selected the first 19 consecutive CT scans with a HSL.”, page 8, line 161-163* |
|  |  | (b) Give reasons for non-participation at each stage *🡪 not applicable with our study design* |
|  |  | (c) Consider use of a flow diagram *🡪 not applicable with our study design* |
| Descriptive data | 14* | (a) Give characteristics of study participants (eg demographic, clinical, social) and information on exposures and potential confounders *🡪 not applicable with our study design since we only compare anatomical characteristics. These are not affected by these data* |
|  |  | (b) Indicate number of participants with missing data for each variable of interest  *🡪 not applicable with our study design* |
|  |  | (c) *Cohort study*—Summarise follow-up time (eg, average and total amount)  *🡪 not applicable with our study design* |
| Outcome data | 15* | *Cohort study*—Report numbers of outcome events or summary measures over time |
|  |  | *Case-control study—*Report numbers in each exposure category, or summary measures of exposure |
|  |  | *Cross-sectional study—*Report numbers of outcome events or summary measures *🡪 results section* |
| Main results | 16 | (*a*) Give unadjusted estimates and, if applicable, confounder-adjusted estimates and their precision (eg, 95% confidence interval). Make clear which confounders were adjusted for and why they were included *🡪 results section* |
|  |  | (*b*) Report category boundaries when continuous variables were categorized  *🡪 not applicable* |
|  |  | (*c*) If relevant, consider translating estimates of relative risk into absolute risk for a meaningful time period  *🡪 not applicable* |
| Other analyses | 17 | Report other analyses done—eg analyses of subgroups and interactions, and sensitivity analyses *🡪 no subgroup analyses done* |
| Discussion | | |
| Key results | 18 | Summarise key results with reference to study objectives *🡪 “The results of this study show that GTFs and HSLs have different anatomical characteristics. The origin, center, endpoint, and height were different for these two lesions. GTFs were more likely to involve the supraspinatus footprint and more likely to be off-track. page 10, line 187-189* |
| Limitations | 19 | Discuss limitations of the study, taking into account sources of potential bias or imprecision. Discuss both direction and magnitude of any potential bias *🡪 “A limitation of this study is that fracture mapping was not possible due to differences in lesion characteristics (e.g. a HSL being an impression fracture and a GTF being a fracture with a displaced greater tuberosity). Therefore, we assessed the location of the HSLs and GTFs in the axial and sagittal plane separately, as described by Cho et al.. 2 Another limitation is that the relatively small sample size was sufficient to detect large (Cohen’s d≈1) differences between groups. 3 More subtle differences may also exist but did not reach statistical significance in this study. Furthermore, the glenoid track concept is not validated for GTF and thus these findings should be interpreted cautiously. Moreover, although all measurements were performed by two investigators, we know that interobserver reliability of CT scan measurements is limited. This could affect reproducibility of our findings. Finally, in our practices we did not perform CT scans for all patients. Since we only included patients with a CT scan retrospectively, this could lead to selection bias..”, page 10, line 190-201* |
| Interpretation | 20 | Give a cautious overall interpretation of results considering objectives, limitations, multiplicity of analyses, results from similar studies, and other relevant evidence 🡪 “When comparing our findings with previous work on this topic, the bicipital angle in our study was a bit higher than reported by Cho et al. 2 (114.6˚ in engaging lesions and 118.8˚ in non-engaging lesions). Since we have followed the same measurement method in Cho et al. this could be due to measurement variation. This difference in absolute angles does not affect the main findings of this study comparing HSL and GTF . Hasan et al. concluded that GTFs are more likely to occur in the zone of the interval between the supraspinatus and infraspinatus footprint. 9 In our study all GTFs involved the infraspinatus tendon footprint and 72% involved the supraspinatus footprint. This corresponds with the results of Hasan et al. regarding that these lesions are mostly located in the interval between the supraspinatus and infraspinatus footprint.  GTFs are associated with a lower recurrent dislocation rate. 5,13 Our results could explain this phenomenon as GTFs are more laterally oriented in comparison with HSL and thus are more likely to be extra-capsular. A second explanation could be that due to the existence of the fracture less force is excreted to the glenoid rim and the capsulolabral structures. A third factor contributing to the lower recurrence rate could be the loss of end-range of motion resulting in less engagement of the fracture with the glenoid. 5 A fourth possible contributing factor could be that these lesions are associated with the middle aged population. 5 Since age is a predicting factor for instability, these patients could be less prone to recurrent instability. 5 This also results in protection from instability by preventing the patient from performing movements leading to instability in end-range of abduction and external rotation.  Three different etiologies are postulated regarding Hill Sachs lesions and greater tuberosity fractures in the setting of glenohumeral dislocation:  1. GTFs and HSLs are both caused by impaction of the humeral head with the glenoid.  2. GTFs are a result of an avulsion of the rotator cuff.  3. GTFs are a combination of an avulsion and an impaction fracture; if the lateral side of the humeral head collides with the glenoid the impact results in a weak spot which aids the rotator cuff to avulse the greater tuberosity.  Our results show that HSLs and GTFs differ in location and therefore we believe that the moment of impact of GTFs and HSL is different. This makes theory 1 that they are both due to an impaction of the humeral head and glenoid unlikely. We observed that GTFs were laterally located. This corresponds with theory 2 that GTF and HS are a result of an avulsion fracture caused by the rotator cuff. Furthermore, it clarifies that fracture patterns are closely related to the rotator cuff attachments. 9 The fact that there were no patients having both a GTF and a HSL supports the theory of a combination of an avulsion and an impaction fracture. In summary, it is likely that most GTFs are avulsion fractures of the rotator cuff. page 11-12 lines 202-237 |
| Generalisability | 21 | Discuss the generalisability (external validity) of the study results 🡪 “When comparing our findings with previous work on this topic, the bicipital angle in our study was a bit higher than reported by Cho et al. 2 (114.6˚ in engaging lesions and 118.8˚ in non-engaging lesions). Since we have followed the same measurement method in Cho et al. this could be due to measurement variation. This difference in absolute angles does not affect the main findings of this study comparing HSL and GTF. Hasan et al. concluded that GTFs are more likely to occur in the zone of the interval between the supraspinatus and infraspinatus footprint. 9 In our study all GTFs involved the infraspinatus tendon footprint and 72% involved the supraspinatus footprint. This corresponds with the results of Hasan et al. regarding that these lesions are mostly located in the interval between the supraspinatus and infraspinatus footprint. ” Page 10-11, lines 202-211 |
| Other information | | |
| Funding | 22 | Give the source of funding and the role of the funders for the present study and, if applicable, for the original study on which the present article is based *🡪 “All authors, their immediate families, and any foundation with which they are affiliated did not receive any financial payments or other benefits from any commercial entity related to the subject of this article.”* page 12, line 245-247 |

*Give information separately for cases and controls in case-control studies and, if applicable, for exposed and unexposed groups in cohort and cross-sectional studies.

**Note:** An Explanation and Elaboration article discusses each checklist item and gives methodological background and published examples of transparent reporting. The STROBE checklist is best used in conjunction with this article (freely available on the Web sites of PLoS Medicine at http://www.plosmedicine.org/, Annals of Internal Medicine at http://www.annals.org/, and Epidemiology at http://www.epidem.com/). Information on the STROBE Initiative is available at www.strobe-statement.org.
